# Nuclear Norm Regularized Deep Neural Network for EEG-Based Emotion Recognition

**DOI:** 10.3389/fpsyg.2022.924793

**Published:** 2022-06-29

**Authors:** Shuang Liang, Mingbo Yin, Yecheng Huang, Xiubin Dai, Qiong Wang

**Affiliations:** ^1^Smart Health Big Data Analysis and Location Services Engineering Lab of Jiangsu Province, Nanjing University of Posts and Telecommunications, Nanjing, China; ^2^State Key Laboratory for Novel Software Technology, Nanjing University, Nanjing, China; ^3^School of Computer Science and Technology, Nanjing Tech University, Nanjing, China; ^4^Guangdong Provincial Key Laboratory of Computer Vision and Virtual Reality Technology, Shenzhen Institutes of Advanced Technology, Chinese Academy of Sciences, Shenzhen, China

**Keywords:** electroencephalography (EEG), emotion recognition, affective brain-computer interface (aBCI), structural information, nuclear norm regularization

## Abstract

Electroencephalography (EEG) based emotion recognition enables machines to perceive users' affective states, which has attracted increasing attention. However, most of the current emotion recognition methods neglect the structural information among different brain regions, which can lead to the incorrect learning of high-level EEG feature representation. To mitigate possible performance degradation, we propose a novel nuclear norm regularized deep neural network framework (NRDNN) that can capture the structural information among different brain regions in EEG decoding. The proposed NRDNN first utilizes deep neural networks to learn high-level feature representations of multiple brain regions, respectively. Then, a set of weights indicating the contributions of each brain region can be automatically learned using a region-attention layer. Subsequently, the weighted feature representations of multiple brain regions are stacked into a feature matrix, and the nuclear norm regularization is adopted to learn the structural information within the feature matrix. The proposed NRDNN method can learn the high-level representations of EEG signals within multiple brain regions, and the contributions of them can be automatically adjusted by assigning a set of weights. Besides, the structural information among multiple brain regions can be captured in the learning procedure. Finally, the proposed NRDNN can perform in an efficient end-to-end manner. We conducted extensive experiments on publicly available emotion EEG dataset to evaluate the effectiveness of the proposed NRDNN. Experimental results demonstrated that the proposed NRDNN can achieve state-of-the-art performance by leveraging the structural information.

## Introduction

Affective brain computer interface (aBCI) can establish an effective communication pathway between brain and devices (Mühl et al., 2014). Emotion recognition enables aBCI to accurately perceive the affective states of brains, which has attracted increasing attention (Fragopanagos and Taylor, 2005). Naturally, there exist several patterns of emotion expression, such as voice signals (Ang et al., 2002), facial expressions (Xiaohua et al., 2017), body gestures (Yan et al., 2014), electromyogram (EMG) signals (Cheng and Liu, 2008), electrocardiogram (ECG) signals (Agrafioti et al., 2012), and electroencephalogram (EEG) signal (Zheng, 2017). Among the above techniques, EEG is the most extensively adopted to record brain electrical activities caused by emotional fluctuations because of its portability and non-invasive way (Liu et al., 2017; Zheng, 2017).

**Figure 1:**
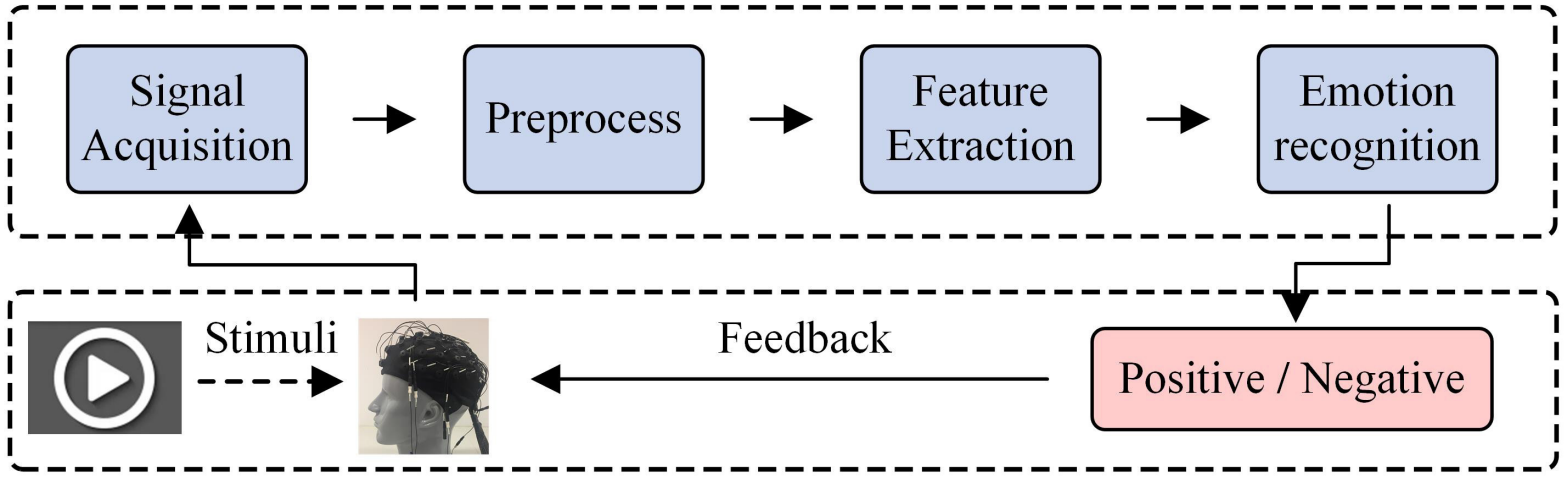
A general EEG-based aBCI.

As shown in Figure 1, a classical EEG-based aBCI system can be divided into four parts (Li J. et al., 2019), namely, signal acquisition, preprocess, feature extraction, and emotion recognition. For the acquisition of EEG signal, the electrical signal of brain activity can be efficiently obtained by the non-invasive electrodes along the scalp. Then, some kinds of filters, e.g., Butterworth and Chebyshev (Bustamante et al., 2015), are adopted to preprocess the original EEG signals to clean the noise. Subsequently, the affective EEG data can be transformed into a suitable feature representation by using the domain-specific feature extractors. In general, the extracted features are mainly represented as follows: (1) time feature; (2) frequency feature; (3) time-frequency feature; and (4) spatial feature. For example, Hjorth Features (Petrantonakis and Hadjileontiadis, 2010) and independent component analysis (ICA) (Iacoviello et al., 2015) are the widely used time domain feature extractors. Wavelet transform (WT) (Mazumder, 2019) and wavelet packet decomposition (WPD) (Ting et al., 2008) are commonly adopted as the affective EEG feature extractors in time-frequency domain. Besides, fast Fourier transform (FFT) (Murugappan and Murugappan, 2013) and autoregressive (AR) model (Atyabi et al., 2016) are two widely used frequency domain affective EEG feature extractors. Common spatial pattern (CSP) is the typical spatial feature extractors for EEG data (Ramoser et al., 2000). The main principle of CSP is to learn an optimal spatial projection, which can maximize the variance of two classes by simultaneous diagonalization of their covariance matrices. Besides the methods mentioned above, many other feature extractors have also been developed for affective EEG feature extraction, such as differential entropy (DE) method (Duan et al., 2013), which is widely used for extracting EEG features in time-spatial domain. After feature extraction, many classifiers can be exploited to emotion recognition, such as support vector machine (SVM) (Zhang et al., 2018), k-nearest neighbor (KNN) (Tang et al., 2019), and Linear Discriminant Analysis (LDA) (Zhang et al., 2013). The recognition results are finally feedback to the users.

Although the abovementioned methods have shown their efficacy in EEG-based aBCI system, these methods belong to shallow learning methods that cannot exploit deep EEG feature representations with the powerful deep learning framework. Therefore, many deep learning methods have been developed for EEG-based emotion recognition. For example, Zheng and Lu (2015) proposed to utilize deep belief network to construct emotion recognition model. Pandey and Seeja (2019) developed a multilayer perceptron-based neural network for EEG-based emotion recognition. In Song et al. (2018), proposed to use graph convolution neural network extract EEG features. Besides, Li Y. et al. (2019) developed a spatial-temporal deep neural network model for emotion recognition. In addition to the above methods, many other deep models have been exploited for decoding EEG, some of them greatly advanced the performance in feature representation and classification. Readers can refer to the in-depth systematic review in Alarcao and Fonseca (2017) for details. Although existing deep learning methods exhibit powerful feature learning capability in dealing with EEG-based emotion data, they have not considered the different contributions of individual brain regions to EEG feature representation and pattern classification.

Recently, neuroscience researches have shown that human emotions are correlated to multiple cerebral cortex regions, such as orbitofrontal cortex and ventromedial prefrontal cortex (Lotfi et al., 2014). Hence, the EEG signals associated with different brain regions might provide different contribution to emotion recognition (Lindquist et al., 2012). In view of this, Li Y. et al. (2019) assigned a set of weights to EEG signals within different brain regions to strengthen or weaken their contributions to EEG decoding. Besides, Park and Chung (2019) selected some good local regions by interquartile range and then adopted local CSP to extract their features. Despite promising progress, most of the current methods do not take into account the reliability of structural information among different brain regions, which can lead to the incorrect learning of high-level EEG feature representation.

Recently, certain methods have been developed to capture the structural information within the EEG feature, such as support matrix machine (SMM) (Luo et al., 2015), Robust SMM (Zheng et al., 2018), and deep stacked SMM (Hang et al., 2020). In Luo et al. (2015), a spectral elastic net regularization was combined with the hinge loss to formulate a matrix classifier, named SMM, which uses nuclear norm to exploit the structural information within EEG feature matrices. Based on SMM, robust SMM (Zheng et al., 2018) was proposed to eliminate outliers within EEG signals and construct a matrix classifier using the recovered clean data. Besides, Hang et al. (2020) adopted SMM as the basic building block to construct a deep stacked SMM, which inherits the characteristic of SMM that can learn the structural information of data as well as the powerful capability of deep representation learning. Although these methods have achieved promise EEG classification performance, they take pre-extracted EEG features as input, which heavily relies on the expertise.

In this study, a novel nuclear norm regularized deep neural network framework (NRDNN) is proposed to capture the structural information among different brain regions in affective EEG decoding. To learn high-level feature representations of multiple brain regions, the proposed NRDNN utilizes different deep neural networks for decoding EEG signals within multiple regions. In view of different brain regions may have different functions for the EEG emotion recognition, NRDNN introduces a region-attention layer to automatically learn weights of different brain regions to strengthen or weaken their corresponding contributions. To leverage the structural information among different brain regions, the weighted feature representations of multiple brain regions are stacked into a feature matrix, the nuclear norm regularization with the hinge loss is used for EEG-based emotion recognition. Besides, NRDNN can be efficiently optimized through standard end-to-end manner. To validate the effectiveness of the proposed method, we conducted extensive experiments on publicly available affective EEG dataset. Experimental results demonstrate that our NRDNN outperforms other comparison methods.

The remainder of this study is organized as follows: the “Nuclear norm regularized deep neural network” section illustrates the proposed NRDNN model and its learning algorithm in detail. In the “Experiments” section, extensive experiments and result analyses are presented. Finally, the conclusions of the study can be found in the “Conclusion” section.

## Nuclear Norm Regularized Deep Neural Network

**Figure 2:**
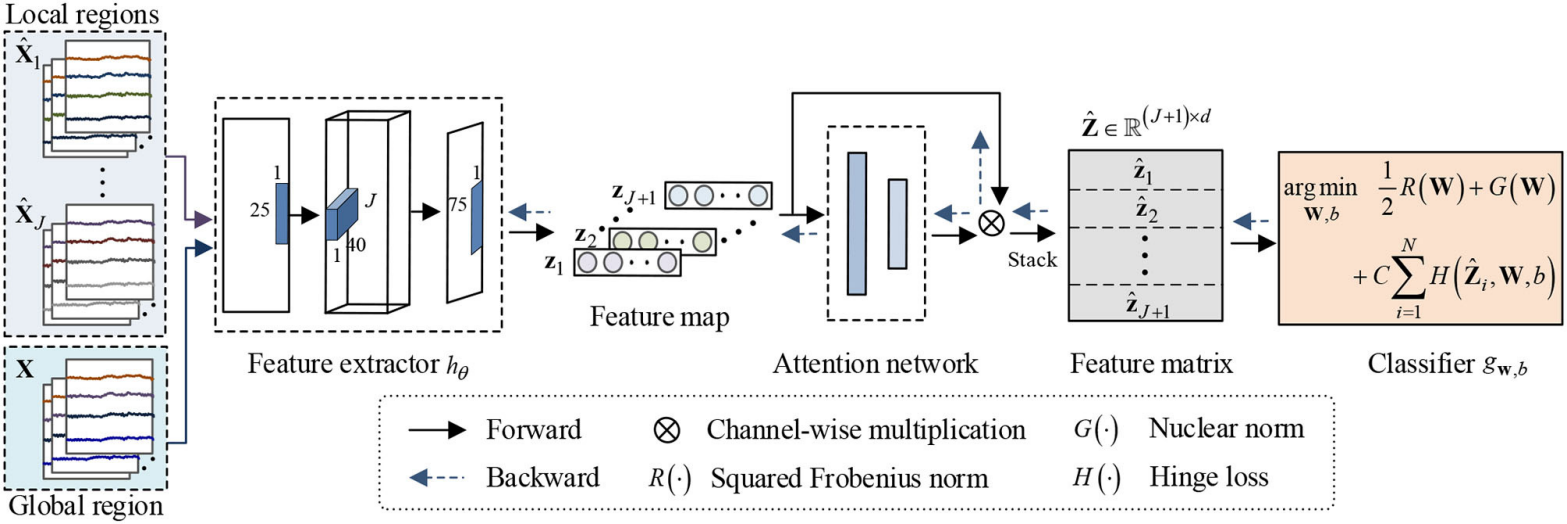
Framework of the proposed NRDNN for EEG-based emotion recognition. NRDNN first utilizes deep neural networks to learn high-level feature representations of multiple brain regions. Then, a set of weights indicating the contributions of each brain region can be automatically learned using a region-attention layer. Subsequently, the weighted feature representations of multiple brain regions are stacked into a feature matrix, and the nuclear norm regularization is adopted to capture the structural information within feature matrix.

Neuroscience studies have shown that relevant information exists among different brain regions (Clark, 1994; Vecchio et al., 2013; Kurmukov et al., 2016). To a certain extent, the structural information among brain regions can reflect this relevant information. Since the nuclear norm of the matrix is the convex approximation of its rank, it can directly capture its structural information between columns or rows. Hence, this study develops an end-to-end nuclear norm regularized deep learning framework by using the structural information in EEG decoding. The flowchart of proposed framework is schematized in Figure 2. As shown in Figure 2, NRDNN first utilizes deep neural networks to learn high-level feature representations of multiple brain regions, respectively. Then, a set of weights indicating the contributions of each brain region can be automatically learned using a region-attention layer. Subsequently, the weighted feature representations of multiple brain regions are stacked into a feature matrix, and the nuclear norm regularization is adopted to capture the structural information within the feature matrix.

### Brain Region EEG Feature Learning

Given a trial of raw affective EEG signal, we aim to indentify the emotion states by decoding this signal. Suppose the EEG signal has *m* channels, each of which has a *t* time point, Thus, the EEG signal can be represented as a two-dimensional matrix $\text{X}=\left[{\text{x}}_{1},{\text{x}}_{2},\cdots \;,{\text{x}}_{m}\right]\in {\mathbb{R}}^{m\times t}$. Here, ${\text{x}}_{i}=\left[x_{1}^{i},x_{2}^{i},\cdots \;,x_{t}^{i}\right]\in {\mathbb{R}}^{t}$ denotes the *i*-th channel of the EEG signal and $x_{j}^{i}$,*j* = 1, 2, ⋯ , *t* denotes the value of the *i*-th channel at the time point *t*.

According to the principle of brain regions (Vecchio et al., 2013), we divide the entire EEG signal **X** into *J* parts located in different brain regions. Without loss of generality, EEG signal located in the *j*-th brain region can be represented as follows:


(1) $$\begin{array}{l} {\hat{\text{X}}}_{j}=\left[{\text{x}}_{1}^{j},{\text{x}}_{2}^{j},\cdots \;,{\text{x}}_{m_{j}}^{j}\right]\in {\mathbb{R}}^{m_{j}\times d},j=1,2,\cdots \;,J. \end{array}$$

Here, we use ${\text{x}}_{k}^{j}$, which represents the EEG signal of the *k*-th channel located in the *j*-th brain region. *m*_*j*_ denotes the number of channels, which is located in the *j*-th brain region. Besides, we have *m*_1_+*m*_2_+⋯+*m*_*J*_ = *m*.

In general, a deep classification model *f* can be decomposed as *f* = *g* ◦ *h*, in which $h_{\theta }:\text{X}\to {\mathbb{R}}^{d}$ parameterized by the network weight θ that maps the input EEG signal **X** to the high-level feature representation space. Besides, $g_{\text{w},b}:\text{Z}\to {\left[0,1\right]}^{K}$ parameterized by the weight **W** and bias *b* that maps the feature representation to the final output.

Currently, many deep neural networks can be used for EEG feature extraction, such as Shallow ConvNet (SConvNet) and Deep ConvNet (DConvNet) developed in Schirrmeister et al. (2017), and EEGNet developed in Lawhern et al. (2018). However, these widely used neural networks focus on motor imagery EEG classification. Hence, we slightly modify SConvNet to form the backbone network for affective EEG decoding (called AConvNet for simplicity), and the detailed network architecture of AConvNet is given in Table 1. To learn deep feature representation of the *j*-th brain region, feature extractor $h_{\theta_{j}}:{\hat{\text{X}}}_{j}\to {\text{z}}_{j}$ parameterized by the network weight θ_*j*_ that maps the EEG signal located in the *j*-th brain region to feature **z**_*j*_. Here, **z**_*j*_, *j* = 1, 2, ⋯ , *J* denotes the deep EEG feature of the *j*-th brain region. In addition, we also apply AConvNet to learn the high-level EEG feature representation of the global brain region, which can be represented as *h*_θ_:**X** → **z**_*J*+1_.

**Table 1:** Network architecture of AConvNet.

**Modules**	**Layers**	**Operation**	**Parameters**	**Size**
Input				*m × d*
Feature extractor	Reshape			1*×m × d*
	Convolution	Conv2D	1 × 25, 40	40*×m ×* (*d*-24)
	Convolution	Conv2D	*m ×* 1, 40	40 × 1 × (*d*-24)
	Normalization	BatchNorm	/	40 × 1 × (*d*-24)
	Activation	Square	/	40 × 1 × (*d*-24)
	Pooling	AveragePool	1 × 75, 15	40 × 1 × [(*d*-99)//
	Activation	log	/	40 × 1 × [(*d*-99)//
	Flatten		/	40 [(*d*-99)15 + 1]
Classification	Fully connected	Dense	40 [(*d*-99)15 + 1] ×300	300
	Fully connected	Dense	300*×K*	*K*

### Discriminative Feature Identification

As pointed earlier, human emotions are correlated to multiple cerebral cortex regions, such as orbitofrontal cortex and ventromedial prefrontal cortex (Lotfi et al., 2014). Hence, the EEG signals acquired from different brain regions would contribute differently to emotion recognition (Lindquist et al., 2012). To identify the contribution of different brain regions, we first reshape all the local and global EEG feature representations into a feature map, which can be represented as follows:


(2) $$\begin{array}{l} \text{Z}=\left[{\text{z}}_{1},{\text{z}}_{2},\cdots \;,{\text{z}}_{J},{\text{z}}_{J+1}\right]\in {\mathbb{R}}^{1\times d\times \left(J+1\right)}. \end{array}$$

**Figure 3:**
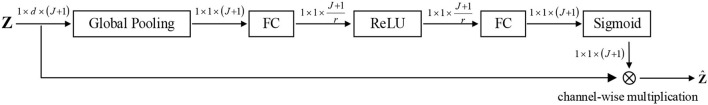
Schema of the region-attention network.

Thus, identification of the contribution of different brain regions equals to assign a set of appropriate weights to *J* + 1 channels. To achieve this goal, we use squeeze-and-excitation (SE) block (Hu et al., 2018) to adaptively emphasize informative channels and suppress the less useful ones, as shown in Figure 3.

To abstract the information of different brain regions, the global average pooling is used to produce channel-wise statistics **s** ∈ ℝ^*J*+1^, which can be obtained by


(3) $$\begin{array}{l} s_{j}=\frac{1}{d}\overset{d}{\underset{i=1}{\sum }}z_{j}^{i},j=1,2,\cdots \;,J+1, \end{array}$$

where $z_{j}^{i}$ and *s*_*j*_ denote the *i*-th and the *j*-th element of **z**_*j*_ and **s**, respectively. To capture the channel-wise dependencies, the following gating operator and an activation is utilized:


(4) $$\begin{array}{l} \text{u}=\sigma \left({\text{W}}_{2}\cdot \delta \left({\text{W}}_{1}\cdot \text{s}\right)\right), \end{array}$$

where δ(·) and σ(·) denote the activation functions ReLU (Nair and Hinton, 2010) and sigmoid, respectively. Besides, ${\text{W}}_{1}\in {\mathbb{R}}^{\frac{J+1}{r}\times \left(J+1\right)}$ and ${\text{W}}_{2}\in {\mathbb{R}}^{\left(J+1\right)\times \frac{J+1}{r}}$. Here, **u** ∈ ℝ^*J*+1^ represents the weights of multiple channels, which can reflect the contributions of brain regions.

Finally, the weighted feature representation of brain region can be repesented as the channel-wise multiplication between the scale *u*_*j*_ and the feature map **z**_*j*_:


(5) $$\begin{array}{l} {\hat{\text{z}}}_{j}=u_{j}\cdot {\text{z}}_{j},j=1,2,\cdots \;,J,J+1, \end{array}$$

where *u*_*j*_ denotes the *j*-th element of **u**.

### Leaning Structural Information

To capture the structural information among multiple brain regions, we first stack the weighted feature representation of multiple brain regions into a feature matrix:


(6) $$\begin{array}{l} \hat{\text{Z}}=\left[{\hat{\text{z}}}_{1},{\hat{\text{z}}}_{2},\cdots \;,{\hat{\text{z}}}_{J},{\hat{\text{z}}}_{J+1}\right]\in {\mathbb{R}}^{\left(J+1\right)\times d}. \end{array}$$

Then, we focus on construct a matrix classifier, i.e., $g_{\text{w},b}:\hat{\text{Z}}\to y$, which can exploit the structural information to help the emotion recognition. Hence, the classifier *g* can be formulated as follows:


(7) $$\begin{array}{l} \underset{\text{W},b}{\text{arg min}}\frac{1}{2}R\left(\text{W}\right)+G\left(\text{W}\right)+C\overset{N}{\underset{i=1}{\sum }}H\left({\hat{\text{Z}}}_{i},\text{W},b\right), \end{array}$$

where **W** ∈ ℝ^(*J*+1) × *d*^ and *b* represent the regression matrix and bias, respectively. *C* > 0 is the trade-off parameter. $R\left(\text{W}\right)=\text{tr}\left({\text{W}}^{T}\text{W}\right)={\left\|\text{W}\right\|}_{F}^{2}$ is the squared Frobenius norm of regression matrix **W**, which can be used to control the complexity of model and avoid the overfitting problem. *G*(**W**) = τ‖**W**‖_*_ denotes the nuclear norm of **W**, where τ > 0 is the penalty parameter. As the convex approximation of the rank of regression matrix **W**, nuclear norm can grasp the structural infromation within the featrue matrix ${\hat{\text{Z}}}_{i},i=1,2,\cdots \;,N$. Besides, we adopt the widely used hinge loss as the loss function because of its ability in sparseness and robustness modeling.


(8) $$\begin{array}{l} H\left({\hat{\text{Z}}}_{i},\text{W},b\right)=\max\left(0,1-y_{n}\cdot \left\{\text{tr}\left({\text{W}}^{T}{\hat{\text{Z}}}_{i}\right)+b\right\}\right). \end{array}$$

Finally, the prediction of test emotion data $\tilde{\text{Z}}$ using classifier *g* can be represented as follows:


(9) $$\begin{array}{l} g\left(\tilde{\text{Z}}\right)=\text{tr}\left({\text{W}}^{T}\tilde{\text{Z}}\right)+b. \end{array}$$

### Optimization

To optimize the parameter of deep classification model *f*, we use the stochastic gradient descent (SGD) method to optimize the objective function in Equation (7), so that the end-to-end training of both feature extractor *h* and classifier *g* can be carried out *via* standard backpropagation. The partial derivatives of the objective function with respect to the regression matrix **W** and bais *b* can be computed efficiently as follows:


(10) $$\begin{array}{l} \nabla_{\text{W}}=\{\begin{array}{c} \frac{1}{2}\cdot \frac{\partial R}{\partial \text{W}}+\frac{\partial G}{\partial \text{W}}\text{,}H\left(\cdot \right)\leq 0\\ \frac{1}{2}\cdot \frac{\partial R}{\partial \text{W}}+\frac{\partial G}{\partial \text{W}}+C\overset{N}{\underset{i=1}{\sum }}\frac{\partial H}{\partial \text{W}}\text{,}H\left(\cdot \right)\leq 0 \end{array} \end{array}$$

(11) $$\begin{array}{l} \nabla_{b}=\{\begin{array}{c} 0\text{,}H\left(\cdot \right)\leq 0\\ C\overset{N}{\underset{i=1}{\sum }}\frac{\partial H}{\partial b}\text{,}H\left(\cdot \right)\leq 0 \end{array} \end{array}$$

where the gradient of nuclear norm ∂*G*/∂**W**could be calculated according to Papadopoulo and Lourakis (2000).

## Experiments

In this section, the proposed NRDNN is evaluated on the publicly available affective EEG datasets [i.e., DEAP dataset (Koelstra et al., 2011)]. The affective EEG dataset are first preprocessed. Then, we introduce the comparison methods and their parameter settings. The experimental results are subsequently presented and discussed in detail. Finally, we conclude this study.

### Affective EEG Data Preparation

The DEAP dataset contains multiple channel physiological signals for analyzing human emotional states. It is composed of 32-channel EEG signals recorded from 32 subjects. The sampling rate is set to 512 Hz. All subjects are required to watch 40 one-min long music video so that their various emotions are stimulated accordingly. Therefore, there are 40 trials per subject, each of which corresponds to affective EEG data stimulated by one music video. After each trial, all subjects are required to perform self-assessments on five dimensions, i.e., valence (from sad to joyful), arousal (from calm to excited), dominance (from submissive to dominant), liking (related to the preference of participants), and familiarity (related to the prior experience of participants). In addition to the rating range of familiarity, which is distributed from 1 (weakest) to 5 (strongest), the remaining dimensions range from 1 to 9. Referring to Yang et al. (2018), we adopt 5 as the threshold of the valence dimension to divide EEG trials into two categories, i.e., if the valence rating is greater (smaller) than 5, it is positive (negative). In this study, we downsampled 32-channel affective EEG signals to 128 Hz. We then bandpass filtered EEG signals between 4 and 45-Hz frequency band. Without loss of generality, we only take the first half subjects to evaluate the effectiveness of the proposed NRDNN, in order to reduce the training time.

The EEG signals in DEAP database were recorded with 32 electrodes following the international 10/20 system. According to the spatial locations of electrodes, we grouped the 32 electrodes into 5 brain regions. Table 2 summarizes the EEG electrodes located in each brain region in detail (Li Y. et al., 2019).

**Table 2:** The EEG electrodes associated with each brain region.

**Brain region**	**Electrode name**
Frontal	Fp1,Fp2,AF3,AF4, F7,F3,Fz,F4,F8
Temporal	T7,T8
Central	FC5,FC1,FC2,FC6,C3,Cz,C4
Parietal	CP1,CP2,CP5,CP6,P7,P3,Pz,P4,P8,PO3,PO4
Occipital	O1,Oz,O2

### Experimental Setup

#### Baseline Methods

In the experiments, the proposed method was compared with the following comparison methods by using the aforementioned affective EEG classification tasks: (1) Support vector machine (SVM) (Zhang et al., 2018), (2) Support matrix machine (SMM) (Luo et al., 2015), (3) EEGNet (Lawhern et al., 2018), (4) Shallow ConvNet for affective EEG (AConvNet) (Schirrmeister et al., 2017), (5) Fusion ConvNet (FConvNet) (Liang et al., 2020), (6) Deep learning with SVM (DLSVM) (Tang, 2013), (7) NRDNN without region-attention layer (DNN), and (8) Our NRDNN.

#### Implementation Details

As the format of the input data of SMM should be matrices, the principal component analysis (PCA) (Placidi et al., 2016) was adopted to reduce the dimension of EEG data to 32 × 16 matrix features. Then, the obtained two-dimensional EEG features were reshaped into vectors, which were used as the input for SVM. Besides, AConvNet was used as the network backbone for FConvNet. Referring to Liang et al. (2020), we took the high-level feature representations of multiple brain regions as multiple views, which were then classified using cross-entropy loss. For DLSVM, we also used AConvNet as its network backbone. The obtained high-level feature representations of multiple brain regions were concatenated and then classified using SVM in an end-to-end manner. The trade-off parameter *C* of SVM, SMM, DLSVM, and our NRDNN was decided through searching from the set {1*e* − 2, 1*e* − 1, 1*e*0, 1*e*1, 1*e*2}. The parameter τ of SMM and our NRDNN was decided through searching from the set {1*e* − 3, 2*e* − 3, 5*e* − 3, 1*e* − 2, 2*e* − 2, 5*e* − 2, 1*e* − 1, 2*e* − 1, 5*e* − 1, 1*e*0}. For all comparison methods, the optimal parameters *C* and τ were chosen by using the 5-fold cross-validation method on the training dataset. For deep learning methods EEGNet, AConvNet, FConvNet, DLSVM, and NRDNN, the batch size and epoch were set to 40 and 1,000, respectively. The learning rate was dynamically changed during optimization using the formula as follows (Pei et al., 2018): $\eta_{p}=\eta_{0}/{\left(1+\alpha p\right)}^{\beta }$, in which *p* linearly changes from 0 to 1, η_0_ = 1*e* − 3, α = 10, and β = 0.75. Besides, the parameter *r* is set to 2 in the region-attention network.

Following the evaluation protocol developed by Lan et al. (2018), we used the leave-one-subject-out cross-validation method to evaluate the affective EEG classification performance on each subject. The following metrics (Chen et al., 2020) on the test dataset were adopted, i.e., Accuracy (ACC), *F*1score (F1), and the area under the receiver operating characteristics curve (AUC). Herein, ACC = (TP+TN)/(TP+FN+FP+TN) and F1 = 2 × PPV × SEN/(PPV+SEN), in which the positive predictive value (PPV) = TP/(TP+FP) and sensitivity (SEN) = TP/(TP+FN). Generally, the higher are the metric values, the better affective is the EEG classification performance.

### Experimental Results Analysis

The classification accuracy (ACC), F1 score (F1), and AUC of all comparison methods on 16 subjects are presented in Tables 3–5. The best classification results are boldfaced. From these classification results, we can obtain the following observations. In terms of ACC, matrix learning method SMM can obtain better classification performance than vector-based classifier SVM. This is because SMM can exploit the correction within EEG feature matrices to improve the classification performance. In addition, deep learning methods, such as EEGNet, AConvNet, FConvNet, DLSVM, and NRCNN, can yield better classification results than shallow methods, such as SVM and SMM, in almost all cases. Compared with shallow methods, deep neural networks can automatically learn high-level feature representations from the raw data, resulting in better EEG decoding performance. It is notable that our NRDNN can obtain the best classification performance than other deep learning methods. The promising results are mainly attributed to the fact that NRDNN can not only learn high-level feature representations of EEG signals located in multiple brain regions but also capture the structural information among different brain regions. The experimental results verify the fact that the structural information among different brain regions is conductive to boost the decoding performance of affective EEG signals.

**Table 3:** Classification performances (ACC) of our NRDNN against the comparison methods.

**Subjects**	**Comparison methods**							
	**SVM**	**SMM**	**EEGNet**	**AConvNet**	**FConvNet**	**DLSVM**	**DNN**	**NRDNN**
*S01*	0.4750	0.5250	0.6250	0.6250	0.7250	0.7280	**0.7500**	**0.7500**
*S02*	0.5500	0.5750	0.6000	0.6250	0.6500	0.7000	0.7000	**0.7250**
*S03*	0.5750	0.6000	0.5500	0.6000	0.6250	0.6375	**0.6500**	**0.6500**
*S04*	0.5500	0.4750	0.6750	0.6750	0.7000	0.7050	0.7250	**0.7500**
*S05*	0.5500	0.7000	0.6500	0.7000	0.7250	0.7250	**0.7750**	**0.7750**
*S06*	0.5500	0.7500	0.7000	0.7250	0.7000	0.7500	**0.7750**	**0.7750**
*S07*	0.5750	0.7000	0.6250	0.7000	0.7250	0.7250	**0.7500**	**0.7500**
*S08*	0.5250	0.6250	0.6000	0.6250	0.6250	0.6000	0.6500	**0.7000**
*S09*	**0.7000**	0.5250	0.6000	0.5750	0.6500	0.6750	0.6500	**0.7000**
*S10*	0.5250	0.6250	0.6250	0.6250	0.6500	0.6750	0.7000	**0.7250**
*S11*	0.4000	0.6500	0.6500	0.6750	0.6500	0.6750	0.7500	**0.7750**
*S12*	0.4750	0.5500	0.6250	0.7000	0.7000	0.7000	0.7000	**0.7250**
*S13*	0.5500	0.5500	0.6500	0.6500	0.7000	0.7000	0.7250	**0.7500**
*S14*	0.5750	0.6000	0.5750	0.5750	0.6000	0.6250	0.6500	**0.7000**
*S15*	0.5000	0.5250	0.6250	0.6500	0.6750	0.6750	**0.7500**	**0.7500**
*S16*	0.5250	0.4000	0.6000	0.6000	0.6250	0.6250	**0.6750**	**0.6750**
*Avg*.	0.5375	0.5859	0.6234	0.6453	0.6703	0.6825	0.7109	**0.7297**

*The best classification results are boldfaced*.

**Table 4:** Classification performances (F1) of our NRDNN against the comparison methods.

**Subjects**	**Comparison methods**							
	**SVM**	**SMM**	**EEGNet**	**AConvNet**	**FConvNet**	**DLSVM**	**DNN**	**NRDNN**
*S01*	0.4747	0.4861	0.5807	0.6248	0.7206	0.7255	0.7469	**0.7500**
*S02*	0.4000	0.4133	0.5657	0.6389	0.6616	0.7222	0.7020	**0.7234**
*S03*	0.5726	0.4984	0.5343	0.5990	0.6248	0.6375	**0.6491**	**0.6491**
*S04*	0.5396	0.4130	0.6484	0.6698	0.6703	0.6918	0.7163	**0.7475**
*S05*	0.5500	0.6581	0.6465	0.6931	0.7163	0.7206	0.7382	**0.7566**
*S06*	0.4872	0.5098	0.6000	0.6204	0.6429	0.6667	0.6257	**0.6894**
*S07*	0.5402	0.4805	0.6190	0.6238	**0.7025**	0.6925	0.6865	0.7024
*S08*	0.5223	0.5636	0.6000	0.6132	0.6190	0.5733	0.6267	**0.6931**
*S09*	0.6970	0.4473	0.5908	0.5248	0.6354	0.6698	0.6419	**0.7000**
*S10*	0.5247	0.5807	0.6229	0.6229	0.6491	0.6647	0.6970	**0.7234**
*S11*	0.3600	0.5333	0.6491	0.6577	0.6419	0.6698	0.7396	**0.7630**
*S12*	0.4667	0.4357	0.6132	0.6992	0.7000	0.6992	0.7000	**0.7234**
*S13*	0.4643	0.5396	0.6011	0.6491	0.6703	0.6970	0.7163	**0.7494**
*S14*	0.5248	0.5442	0.5616	0.5726	0.5908	0.5943	0.6465	**0.6800**
*S15*	0.4885	0.3866	0.6229	0.6491	0.6577	0.6647	**0.7500**	0.7494
*S16*	0.5175	0.3750	0.5833	0.5833	0.3846	0.4398	**0.6647**	**0.6647**
*Avg*.	0.5081	0.4916	0.6025	0.6276	0.6430	0.6581	0.6905	**0.7166**

*The best classification results are boldfaced*.

**Table 5:** Classification performances (AUC) of our NRDNN against the comparison methods.

**Subjects**	**Comparison methods**							
	**SVM**	**SMM**	**EEGNet**	**AConvNet**	**FConvNet**	**DLSVM**	**DNN**	**NRDNN**
*S01*	0.3885	0.3985	0.4862	0.5238	0.6241	0.6479	0.6717	**0.6892**
*S02*	0.2146	0.2652	0.3409	0.5126	0.5000	0.5379	0.5732	**0.6010**
*S03*	0.4520	0.3409	0.3972	0.4596	0.4975	0.4950	0.5177	**0.5202**
*S04*	0.4167	0.2839	0.5208	0.5651	0.5339	0.5776	0.6042	**0.6198**
*S05*	0.4714	0.5417	0.5859	0.6120	0.6380	0.6484	0.6250	**0.6667**
*S06*	0.3400	0.2667	0.4233	0.4533	**0.5533**	0.5300	0.5400	0.5467
*S07*	0.4524	0.3512	0.6012	0.4970	**0.6399**	**0.6399**	0.6250	0.6190
*S08*	0.4116	0.3965	0.4722	0.4722	0.4874	0.4091	0.4899	**0.5732**
*S09*	0.5600	0.3425	0.4725	0.4275	0.4950	0.5025	0.5500	**0.5925**
*S10*	0.3500	0.3775	0.4650	0.4675	0.5000	0.5225	0.5800	**0.6125**
*S11*	0.1563	0.2630	0.4661	0.4479	0.4583	0.4974	0.5677	**0.6042**
*S12*	0.3058	0.2281	0.4236	0.5639	0.5564	0.5539	0.5664	**0.5990**
*S13*	0.2864	0.4271	0.4348	0.5703	0.5217	0.5703	0.5985	**0.6496**
*S14*	0.3500	0.3600	0.3900	0.4225	0.4375	0.4175	0.5300	**0.5375**
*S15*	0.2725	0.1350	0.4200	0.4600	0.4550	0.4700	**0.6000**	0.5800
*S16*	0.4747	0.4027	0.5120	0.5387	0.3947	0.4400	**0.6613**	0.6480
*Avg*.	0.3689	0.3363	0.4632	0.4996	0.5183	0.5287	0.5813	**0.6037**

*The best classification results are boldfaced*.

As summarized in Tables 3–5, we can observe that the proposed method obtains the highest average classification results. Specifically, the proposed NRDNN achieves promising average results of 72.97, 71.66, and 60.37% in terms of ACC, F1, and AUC. Compared with the baseline SVM, the absolute average of ACC, F1, and AUC increases by 19.22, 20.85, and 23.48%, respectively. NRDNN outperforms the results of SMM by an average of 14.38, 22.5, and 26.74%, which validates the high-level feature learning capability of our NRDNN. Compared with DLSVM that does not leverage the structural information among multiple brain regions, the average classification results of NRDNN are increased by 4.72, 5.85, and 7.5% in terms of ACC, F1, and AUC, respectively. Besides, NRDNN outperforms EEGNet by 10.63, 11.41, and 14.5%, and yields 8.44, 8.9, and 10.41% higher average classification results than AConvNet. NRDNN is superior to FConvNet by 5.94, 7.36, and 8.54% in terms of ACC, F1, and AUC, respectively. Furthermore, NRDNN outperforms the results of DNN by an average of 1.88, 2.61, and 2.24%, which validates the contributions of different brain regions that can be automatically identified by our NRDNN. These experimental results demonstrate the effectiveness of the proposed NRDNN.

## Discussion

To evaluate the statistical significance of the experimental results, we further perform pairwise two-tailed *t*-test (Zheng et al., 2018) to verify whether there exist significant differences with a confidence level of 95% between the proposed NRDNN and the comparison methods. The statistical significance comparisons of ACC and F1 of NRDNN and other comparison methods are given in Table 6. The *p*-value less than 0.05 expresses that significant differences exist between the proposed NRDNN and the comparison methods. We highlighted the *p*-values that are less than 0.05 in boldface. As summarized in Table 6, we can see that the null hypothesis can be rejected with 95% confidence level in each case. The statistical results verify that the proposed NRDNN significantly outperformed the comparison methods. This further indicated the capability of the NRDNN to exploit the structural information among multiple brain regions, as well as the powerful high-level affective EEG feature learning capability. The above experimental results illustrate that the proposed NRDNN is suitable for the classification of affective EEG data.

**Table 6:** Statistical significance comparisons of ACC and F1 of NRDNN and other comparison methods.

**Metrics**	**NRDNN vs. SVM**	**NRDNN vs. SMM**	**NRDNN vs. EEGNet**	**NRDNN vs. AConvNet**	**NRDNN vs. FConvNet**	**NRDNN vs. DLSVM**	**NRDNN vs. DNN**
ACC	1.71E-07	3.57E-06	8.18E-13	1.09E-08	5.63E-07	5.75E-06	1.50E-04
F1	2.84E-07	1.06E-08	1.65E-11	2.305E-08	3.11 E-05	8.00E-05	2.20E-05
AUC	4.59E-07	1.93E-09	5.75E-08	3.97E-08	1.16E-05	1.31E-05	4.14E-04

**Figure 4:**
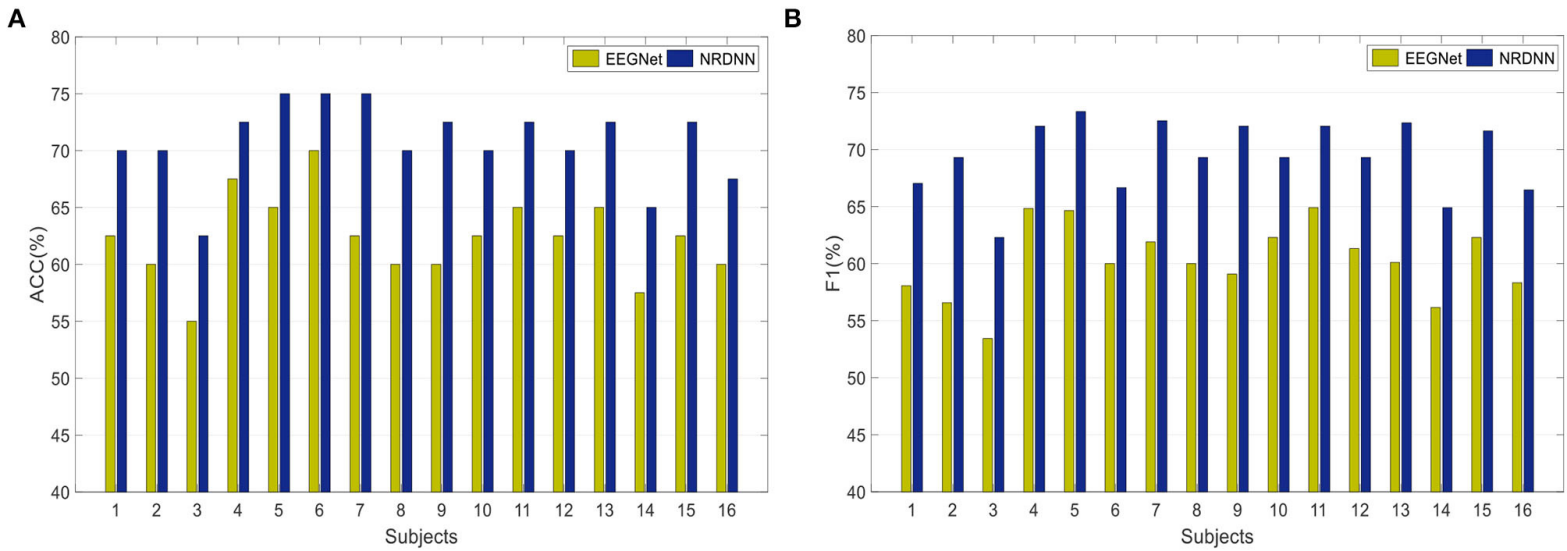
Classification results of both EEGNet and the proposed framework NRDNN using EEGNet as its network backbone. **(A)** Classification performance (ACC), and **(B)** Classification performance (F1).

To obtain a better insight into the classification result of our nuclear norm regularized deep neural network framework, we further investigated the effects of different network backbones on the classification performance. Figure 4A presents the ACCs of both EEGNet and the proposed framework NRDNN using EEGNet as its network backbone. Figure 4B gives the F1s. It can be found that NRDNN yields better results than the baseline EEGNet in all cases. In terms of ACC, NRDNN is superior to EEGNet by 7.5, 10, 7.5, 5, 10, 5, 12.5, 10, 12.5, 7.5, 7.5, 7.5, 7.5, 7.5, 10, and 7.5% on 16 subjects, respectively. Compared to EEGNet that does not leverage the deep features of multiple brain regions and their structural information, the classification F1s of NRDNN are increased by 8.96, 12.74, 8.86, 7.22, 8.68, 6.67, 10.63, 9.31, 12.98, 7.02, 7.15, 7.99, 12.23, 8.75, 9.34, and 8.14%, respectively. The average ACC and F1 of NRDNN are 70.78 and 69.41%. The absolute values are increased by 8.44 and 9.16% compared with EEGNet.

Overall, the proposed NRDNN improves the affective EEG classification performance using different network backbones. The abovementioned results validate that NRDNN can effectively learn deep features of multiple brain regions and their corresponding structural information using the nuclear norm regularization. To summarize, NRDNN integrates the powerful deep feature learning capability and the structural information learning ability of matrix classifier. The experimental results demonstrate that the proposed NRDNN framework could achieve better classification performance than the comparison methods.

## Conclusion

In this study, we first presented a deep neural network, named AConvNet, for affective EEG decoding. Based on AConvNet, we further proposed a novel nuclear norm regularized deep neural network framework called NRDNN. The proposed NRDNN can effectively learn high-level feature representations of EEG signals located in multiple brain regions using AConvNet, as well as discriminate the contributions of multiple brain regions using a set of automatically learned weights. Besides, NRDNN can exploit the structural information among multiple brain regions using the introduced nuclear norm regularization. The proposed NRDNN can be carried out in an efficient end-to-end fashion. Extensive experimental results on publicly available emotion dataset demonstrate the superiority of our NRDNN.

Despite the promising classification performance of NRDNN, there is still room for further improvement. For example, the development of more advance attention mechanism is conductive to the identification of the contribution of different brain regions. Besides, extending the proposed method to multi-class classification is another interesting direction. Furthermore, more powerful discriminative high-level features with both spatial and temporal information of EEG signals can further improve the performance of EEG-based emotion recognition. We will address these issues in the future studies.

## Data Availability Statement

Publicly available datasets were analyzed in this study. This data can be found at: http://www.eecs.qmul.ac.uk/mmv/datasets/deap/download.html.

## Author Contributions

SL is responsible for study design and manuscript writing. MY and YH are responsible for data processing and data analysis. XD is responsible for manuscript editing. QW is responsible for experimental design. All authors contributed to the article and approved the submitted version.

## Funding

This study was supported in part by the National Natural Science Foundation of China under Grants (61902197). Research on development and application of intelligent vehicle testing simulator based on augmented reality was funded by the Guangdong Provincial Basic and Applied Basic Research Fund-Regional Joint Fund (2020B1515130004), the Open Project of State Key Laboratory for Novel Software Technology at Nanjing University (KFKT2020B11), and the NUPTSF (NY219034).

## Conflict of Interest

The authors declare that the research was conducted in the absence of any commercial or financial relationships that could be construed as a potential conflict of interest.

## Publisher's Note

All claims expressed in this article are solely those of the authors and do not necessarily represent those of their affiliated organizations, or those of the publisher, the editors and the reviewers. Any product that may be evaluated in this article, or claim that may be made by its manufacturer, is not guaranteed or endorsed by the publisher.
